# Molecular Analysis of Prothrombotic Gene Variants in Venous Thrombosis: A Potential Role for Sex and Thrombotic Localization

**DOI:** 10.3390/jcm9041008

**Published:** 2020-04-02

**Authors:** Gustavo Cernera, Alessandro Di Minno, Felice Amato, Ausilia Elce, Renato Liguori, Dario Bruzzese, Antonella Miriam Di Lullo, Giuseppe Castaldo, Federica Zarrilli, Marika Comegna

**Affiliations:** 1 Dipartimento di Medicina Molecolare e Biotecnologie Mediche, Università di Napoli Federico II, 80131 Naples, Italy; cernera@ceinge.unina.it (G.C.); felice.amato@unina.it (F.A.); liguorir@ceinge.unina.it (R.L.); giuseppe.castaldo@unina.it (G.C.); marika.comegna@unina.it (M.C.); 2 CEINGE-Biotecnologie avanzate, 80131 Naples, Italy; alessandro.diminno@unina.it (A.D.M.); elce@ceinge.unina.it (A.E.); antonella.dilullo@libero.it (A.M.D.L.); 3 Dipartimento di Farmacia, Università di Napoli Federico II, 80131 Naples, Italy; 4 Università Telematica Pegaso, 80143 Naples, Italy; 5 Dipartimento di Sanità Pubblica, Università di Napoli Federico II, 80131 Naples, Italy; dario.bruzzese@unina.it; 6 Dipartimento di Neuroscienze e Scienze Riproduttive ed Odontostomatologiche, Università di Napoli Federico II, 80131 Naples, Italy

**Keywords:** pulmonary embolism, vein thrombosis, portal vein thrombosis, retinal vein thrombosis, cerebral vein thrombosis, prothrombotic variants

## Abstract

*Background:* Requests to test for thrombophilia in the clinical context are often not evidence-based. *Aim:* To define the role of a series of prothrombotic gene variants in a large population of patients with different venous thromboembolic diseases. *Methods*: We studied Factor V Leiden (FVL), FVR2, FII G20210A, Methylenetetrahydrofolate reductase (MTHFR) C677T and A1298C, beta-fibrinogen -455 G>A, FXIII V34L, and HPA-1 L33P variants and PAI-1 4G/5G alleles in 343 male and female patients with deep vein thrombosis (DVT), 164 with pulmonary embolism (PE), 126 with superficial vein thrombosis (SVT), 118 with portal vein thrombosis (PVT), 75 with cerebral vein thrombosis (CVT) and 119 with retinal vein thrombosis (RVT), and compared them with the corresponding variants and alleles in 430 subjects from the general population. *Results:* About 40% of patients with DVT, PE and SVT had at least one prothrombotic gene variant, such as FVL, FVR2 and FII G20210A, and a statistically significant association with the event was found in males with a history of PE. In patients with a history of PVT or CVT, the FII G20210A variant was more frequent, particularly in females. In contrast, a poor association was found between RVT and prothrombotic risk factors, confirming that local vascular factors have a key role in this thrombotic event. *Conclusions:* Only FVL, FVR2 and FII G20210A are related to vein thrombotic disease. Other gene variants, often requested for testing in the clinical context, do not differ significantly between cases and controls. Evidence of a sex difference for some variants, once confirmed in larger populations, may help to promote sex-specific prevention of such diseases.

## 1. Introduction

Venous thromboembolism (VTE) includes deep vein thrombosis (DVT) and pulmonary embolism (PE), which often appears as a complication of DVT. Recently, thrombophlebitis was classified as superficial vein thrombosis (SVT) and was recognized as a high-risk condition for PE [1]. The mechanisms and predisposing conditions for unusual thrombosis localizations, e.g., cerebral [2], portal [3] and retinal vein localizations [4], may differ from the ones identified for VTE [5]. VTE is the third most frequent cause of cardiovascular diseases and death, and it represents a relevant medical and social problem for its high occurrence and the severity of the phenotype in a large percentage of cases [6,7]. VTE may be prevented and effectively treated; therefore, the search for risk factors is a major practical objective [8].

Venous thromboembolism is a multifactorial condition that results from the co-existence of acquired and/or inherited predisposing factors acting cumulatively, including some clinical conditions, such as autoimmune and inflammatory disorders, inflammatory bowel disease and celiac disease [9], or risk factors such as an altered body mass index, platelet and white blood cell count. Hypercoagulable states, i.e., variants of genes involved in hemostasis displaying pro-coagulant effects, play a pivotal role in this respect [5].

Among these, Factor V Leiden (FVL, R506Q variant) causes resistance of FV to protein C inhibition; the G20210A variant of prothrombin enhances the synthesis and the activity of Factor II (FII), and the C677T variant of methylene-tetrahydrofolate reductase (MTHFR) impairs the activity of the enzyme, causing an increase in serum homocysteine. In addition to these well-known prothrombotic gene variants, other variants that are thought to play a role in some forms of venous thrombosis include FV H1299R (FVR2), MTHFR A1298C, factor XIII (FXIII) V34L, human platelet antigen (HPA)-1 L33P, beta-fibrinogen -455G>A and plasminogen activator inhibitor (PAI)-1 4G/5G variants [5,10]. Inconclusive results are present in the literature regarding the role of such variants as risk factors for venous thrombosis. This inconsistency is supposed to be due to (i) the heterogeneous criteria used to select the patients in previous studies; (ii) the fact that most studies pooled male and female patients, with sex differences being potentially hidden [7,11]; (iii) the under-representation of women in most studies [12]; and (iv) the fact that some studies compared the frequency of prothrombotic gene variants in patients and controls from different geographical areas.

Our aim was to test a series of prothrombotic gene variants in a large population of patients with various forms of venous thromboembolic diseases to define their frequencies (as well as determine their frequencies in the different sexes) and compare them with the corresponding frequencies in subjects from the general population from the same geographic area (southern Italy).

## 2. Materials and Methods

### 2.1. Patients

The study was approved by the Ethical Committee of the University Federico II, Naples, Italy (protocol n. 370/18) and was conducted in accordance with the Helsinki Declaration. All clinical and laboratory data were anonymized. Our laboratory acts as the reference lab for molecular diagnostics in the Campania region (southern Italy, about 5 million inhabitants). During the last twelve years (i.e., 2007–2018), we received thousands of requests for the molecular analysis of thrombophilia for different diseases. For this study, we retrospectively analyzed the results obtained in patients referred as affected by various forms of venous thrombotic diseases and compared them with a group of subjects from the general population (GP) from the same geographical area (Campania region, southern Italy).

For each subject, we recorded all anagraphical and clinical data and verified that the diagnosis of each disease had been performed according to current guidelines. Exclusion criteria: Patients with a doubtful diagnosis were excluded from the study. In addition, for cases in which multiple patients belonged to the same family, we considered only the first case temporally analyzed. Patients under anticoagulant therapy were not excluded from the study since genetic analysis is not influenced by such therapies. In detail, our study population included the following subjects:

(i) 430 subjects from the GP who, at the personal anamnesis (collected by a physician trained in the field of thromboembolic diseases), did not describe any episodes of venous thrombosis (median age: 43 years; range: 5–85 years; 265 females); exclusion criteria: all cases in which a previous thromboembolic event was suspected;
(ii) 343 patients who experienced at least one episode of DVT (median age: 53 years; range: 10–92 years; 172 females) diagnosed by D-dimer analysis, followed by ultrasound (US) or computed tomography (CT) when necessary [13];
(iii) 164 patients who experienced at least one episode of PE (median age: 56 years; range: 12–93 years; 101 females); the diagnostic protocol for PE conformed to the 2014 European Society of Cardiology (ESC) guidelines [14];
(iv) 126 patients who experienced at least one episode of SVT (median age: 53 years; range: 16–79 years; 81 females);
(v) 118 patients who experienced at least one episode of PVT as a complication of chronic liver disease (median age: 54 years; range: 6–79 years; 52 females); the diagnosis was confirmed by different instrumental approaches [15];
(vi) one episode of cerebral vein thrombosis (CVT) (median age: 40 years; range: 1–76 years; 54 females) diagnosed by magnetic resonance (MR) (80% of cases) or CT venography [16];
(vii) 119 patients who experienced at least one episode of retinal vein thrombosis (RVT) (median age: 55 years; range: 5–88 years; 65 females) confirmed by fluoro-angiography and optical coherence tomography (OCT) angiography in about a half of cases [17].

### 2.2. DNA Extraction

Blood samples were collected by venipuncture into EDTA tubes using the Vacutainer system. DNA was extracted from leukocytes using a commercial automated procedure (Roche, Italy). The DNA was spectrophotometrically quantified (also to verify the purity) and analyzed for FVL (R506Q variant); FVR2 (H1299R variant); FII, G20210A; MTHFR, C677T and A1298C; beta-fibrinogen, -455 G>A; FXIII, V34L; HPA-1, L33P variants and PAI-1 4G/5G alleles. All the variants were analyzed using a LightCycler 1.2 Instrument, (Roche Diagnostics, Basel, Switzerland), which uses PCR for the amplification of the genomic region of interest and fluorogenic target-specific hybridization for the detection and genotyping of the amplified DNA, according to the manufacturer’s procedures (Roche Diagnostics). Finally, the results were analyzed using LightCycler Software 3.5 (Roche Diagnostics, Basel, Switzerland) [18].

### 2.3. Statistical Analysis

Both allele and genotype frequencies are reported as absolute numbers and percentages. Differences among groups were accordingly assessed using the Chi-square test. Moreover, in order to quantify the effect of each variant on the disease risk, univariate odds ratios (ORs) with their corresponding 95% confidence intervals (95% CIs) were computed. Multivariable logistic regression models were built to assess whether age and sex could act as effect modifiers of the variant–disease association. In cases of a significant interaction, ORs were computed separately for each sex and each age group. Statistical analyses were performed with the statistical platform [4].

## 3. Results

### 3.1. General Population.

The allele and genotype frequencies of each gene variant are displayed in Table 1 (panel A) and 2 (column: GP). No differences were observed between males and females in allele or genotype frequency (Table 1 panel B, column GP).

### 3.2. Deep Vein Thrombosis, Pulmonary Embolism and Superficial Vein Thrombosis.

The allele frequency of FVL, FVR2 and FII G20210A variants was significantly higher in patients with DVT, PE or SVT than in the subjects from the GP. This was reflected by a different distribution of the genotype frequencies of FVL, FVR2 and FII G20210A variants between the general population and patients with DVT, with PE and with SVT (Table 2). In each case, a statistically significant increase in the odds ratios for the presence of at least one of the three variants (but not for all the other gene variants, Table 1 panel A) was found in subjects with a history of such thrombotic events (Table 3). A significant interaction was observed for the FVL variant: an almost six-fold increase in the odds of PE in male patients (but not in female patients) was found (Table 3). Likewise, the allele frequencies of FVL and FVR2 were higher in males than in females with DVT, PE or SVT, but it was statistically significant only in PE subjects (Table 1 panel B). For each of the variants (Table 3), no significant interaction was observed with age (first episode before/after 50 years of age) in DVT, PE and in SVT patients.

### 3.3. Portal Venous Thrombosis.

The allele (Table 1 panel A) and genotype (Table 2) frequencies of FVL and the FII G20210A variants were significantly different between patients with PVT and the GP, leading to a significant increase in the odds of PVT associated with these two variants (Table 4 panel A). For all the other variants, no difference was observed between patients with PVT and the GP. No differences or interactions according to sex and age were revealed by the analysis (Table 4A).

### 3.4. Cerebral Venous Thrombosis.

As shown in Table 1 (panel A) and Table 2, the allele and genotype frequencies of the FII G20210A variant significantly differed between patients with CVT and the GP, leading to a significant increase in the odds of CVT associated with this variant (Table 4 panel B). No significant interactions with sex and age were observed for any of the variants (Table 4 panel B); only the frequency of the FII G20210A variant was higher (although not significantly) in females (Table 1 panel B).

### 3.5. Retinal Venous Thrombosis.

Allele (Table 1 panel A) and genotype (Table 2) frequencies were entirely comparable between patients with RVT and the GP. No significant interactions with sex and age were observed for these variants.

## 4. Discussion

Pulmonary embolism, DVT and SVT are known to share a genetic predisposition [5,6,7,8]. In fact, in patients with such diseases, the allele frequency of FVL, FVR2 and FII G202010A was significantly higher. Interestingly, in the present setting, the allele frequency of FVL and FVR2 was significantly higher in males than in females with PE. A sex difference in the allele frequency of prothrombotic variants in patients with venous thrombosis, once confirmed in large populations, may affect the strategies for care and prevention since the mechanisms of sex difference in the epidemiology of thrombotic diseases are related to yet-unknown factors [10]. A sex difference in the allele frequency of FVL and FII G20210A variants was previously found in females with juvenile acute myocardial infarction (AMI), and in males with AMI [11]. Most previous studies did not find/assess sex differences for prothrombotic variants in patients with a history of venous or arterial thrombotic disorders [12]. However, in an Iranian population with VTE, Farajzadeh et al. found a significantly higher frequency of the FII G20210A and PAI-1 4G alleles in females [8]. In the present study, no sex difference was identified in the general population for any of the variants examined.

The frequencies of all other prothrombotic gene variants were not significantly different between patients with VTE and the GP in the present study. In agreement with our results, two studies ruled out beta-fibrinogen -455 A>G [12,19] and the FXIII V34L [20] as risk factors for VTE. Similarly, FXIII V34L, beta-fibrinogen -455, HPA-1, MTHFR C677T and A1298C variants and PAI-1 4G/5G alleles did not demonstrate a relationship with VTE [21]. At variance with the present data are previous studies that found MTHFR A1298C alone [22] or in combination with the C677T variant to be associated with VTE [23]. Likewise, a significant increase in the PAI-1 4G allele frequency was found in VTE patients who also had FVL [24]. Differences in sample size and geography may well explain the discrepancies between these reports and the present data.

In contrast to the results of Parik et al. [25], the presented data show that FVL and FII G20210A variants appear to be more frequent in patients with portal vein thrombosis as compared with the GP. This agrees with a previous study [4]. We would like to emphasize that all cases with portal vein thrombosis in the present patient group had chronic liver disease: inflammation and fibrosis cause an increase in intrahepatic vascular resistance, while the alteration of local circulation causes reduced portal flow velocity. These two alterations are the major pathogenic cause of portal hypertension in patients with chronic liver disease [26,27].

In agreement with previous reports [28,29], our results show a significantly higher than normal frequency of FII G20210A in patients with cerebral venous thrombosis. At variance with older data [22,23], however, the presented data rule out a role for MTHFR and FVL variants in CVT. Interestingly, in CVT (as we observed in PVT), the frequency of FII G20210A is higher in females than in males. Cerebral venous thrombosis is common in young subjects and is about three times more common in females than in males [28]. Our results confirm this information and support a role for hormones in this setting [30]. Overall, the role of FII G20210A, particularly in females with CVT, seems to deserve further study with larger populations.

Finally, prothrombotic gene variants have a minor role as risk factors for retinal vein thrombosis in the present setting. This is in keeping with the concept that, in contrast to other forms of VTE, thrombophilia (including FVL and G20210A) seems to be marginal in RVT [26,31,32]. Routine testing for prothrombotic variants appears to be of little value in patients with RVT [32].

The present data argue that prothrombotic gene variants have a role in DVT, PE and SVT, with about 40% of patients showing at least one of the FVL, FVR2 or FII G20210A variants. Such predisposition is significantly more pronounced in males, confirming that sex-related risk factors act in VTE. Portal, cerebral and retinal vein thromboses seem to be less related to prothrombotic gene variants, and only FII G20210A is relevant, particularly in females, again confirming the existence of sex-related risk factors. The evidence of a sex-related difference in some prothrombotic variants appears to be a major direction to be pursued in large population studies to help design sex-specific strategies for thrombosis prevention. In future studies, it would be useful to evaluate, in addition to prothrombotic gene variants, other risk factors for thrombotic events, such as platelet number and BMI, and prospectively monitor patients with VTE in order to relate these factors to the risk of recurrence.

## 5. Conclusions

Of the gene variants frequently requested for testing in the clinical context, only FVL, FVR2 and FII G20210A appear to be related to vein thrombotic disease in the present report. Improving the appropriateness of laboratory test requests improves benefits to the patient and the national health system.

## Figures and Tables

**Table 1:** Allele frequency, *n* (%), of the 9 prothrombotic gene variants. (**A**) Allele frequency in deep vein thrombosis (DVT, *n* = 343), pulmonary embolism (PE, *n* = 164), superficial vein thrombosis (SVT, *n* = 126), portal vein thrombosis (PVT, *n* = 118), cerebral vein thrombosis (CVT, *n* = 75), retinal vein thrombosis (RVT, *n* = 119) and the general population (GP, *n* = 430). (**B**) Allele frequency of FV R506Q, FV H1299R and FII G20210A variants in males and females. * *p* < 0.001; ** *p* < 0.01; *** *p* < 0.05 (*p* value by Chi-square test).

A (General Population)							
	GP	DVT	PE	SVT	PVT	CVT	RVT
FV R506Q (FVL)	21 (2.4)	60 (8.8) *	22 (6.7) *	22 (8.7) *	15 (6.4) **	4 (2.7)	9 (3.8)
FV H1299R (FVR2)	41 (5.0)	58 (8.5) **	31 (9.5) **	25 (9.9) **	16 (6.9)	11 (7.3)	10 (4.2)
FII G20210A	23 (2.7)	53 (7.7) *	27 (8.2) *	14 (5.6) ***	25 (10.6) *	16 (10.7) *	8 (3.4)
MTHFR C677T	376 (43.8)	294 (42.9)	148 (45.1)	120 (48.0)	103 (43.6)	68 (45.9)	101 (42.4)
MTHFR A1298C	249 (29.0)	166 (28.7)	108 (32.9)	64 (25.4)	79 (33.5)	43 (29.1)	53 (22.5)
beta-fibrinogen -455G>A	171 (19.9)	126 (20.6)	65 (19.8)	56 (22.4)	49 (20.8)	37 (25.0)	48 (20.2)
FXIII V34L	153 (17.8)	120 (17.5)	53 (16.1)	35 (13.9)	39 (16.5)	26 (17.3)	42 (17.7)
HPA-1 L33P	128 (14.9)	83 (14.0)	54 (16.5)	49 (19.4)	28 (11.9)	30 (20.0)	28 (11.8)
PAI-1 4G/5G	399 (46.4)	235 (40.7)	166 (50.6)	107 (42.8)	115 (48.7)	61 (40.7)	112 (47.1)
**B (Patients)**							
**FV R506Q (FVL)**							
males	7/328 (2.1)	37/338 (10.9)	13/124 (10.5) ***	9/90 (10.0)	9/132 (6.8)	1/42 (2.4)	6/108 (5.6)
females	14/530 (2.6)	23/344 (6.7)	9/202 (3.5)	13/162 (8.0)	6/104 (5.8)	3/108 (2.8)	3/130 2.3)
**FV H1299R (FVR2)**							
males	16/314 (5.1)	31/338 (9.2)	17/124 (13.7) ***	11/90 (12.2)	11/132 (8.3)	2/42 (4.8)	6/106 (5.7)
females	24/500 (4.8)	27/344 (7.8)	14/202 (6.9)	14/162 (8.6)	5/104 (4.8)	9/108 (8.3)	4/130 (3.1)
**FII G20210A**							
males	9/326 (2.8)	30/338 (8.9)	11/124 (8.9)	8/90 (8.9)	10/132 (7.6)	2/42 (4.8)	2/108 (1.9)
females	14/526 (2.7)	23/344 (6.7)	16/202 (7.9)	7/162 (4.3)	15/104 (14.4)	14/108 (13.0)	6/130 (4.6)

**Table 2:** Genotype frequency, *n* (%), of FV R506Q, FV H1299R and FII G20210A variants. Comparison between deep vein thrombosis (DVT, *n* = 343), pulmonary embolism (PE, *n* = 164), superficial vein thrombosis (SVT, *n* = 126), portal vein thrombosis (PVT, *n* = 118), cerebral vein thrombosis (CVT, *n* = 75), retinal vein thrombosis (RVT *n* = 119) and the general population (GP, *n* = 430). * *p* < 0.001; ** *p* < 0.01; *** *p* < 0.05 (*p* value by Chi-square test).

	GP	DVT	PE	SVT	PVT	CVT	RVT
FV R506Q (FVL)							
RR	409 (95.1)	287 (83.7) *	144 (87.8) **	104 (82.5) *	103 (87.3) **	71 (94.7)	109 (92.4)
RQ	21 (4.9)	52 (15.2)	18 (11.0)	22 (17.5)	15 (12.7)	4 (5.3)	9 (7.6)
QQ	0	4 (1.2)	2 (1.2)	0	0	0	0
**FV H1299R (FVR2)**							
HH	369 (90.4)	285 (83.1) **	133 (81.1) **	101 (80.2) **	102 (86.4)	64 (85.3)	108 (91.5)
HR	37 (9.1)	58 (16.9)	31 (18.9)	25 (19.8)	16 (13.6)	11 (14.7)	10 (8.5)
RR	2 (0.5)	0	0	0	0	0	0
**FII G20210A**							
GG	404 (94.6)	294 (85.7) *	139 (84.8) *	112 (88.9) ***	94 (79.7) *	59 (78.7) *	111 (93.3)
GA	23 (5.4)	45 (13.1)	23 (14.0)	14 (11.1)	23 (19.5)	16 (21.3)	8 (6.7)
AA	0	4 (1.2)	2 (1.2)	0	1 (0.8)	0	0

**Table 3:** Odds ratios and confidence intervals of the FV R506Q, FV H1299R and FII G20210A variants. (**A**) Deep venous thrombosis (DVT, *n* = 343), (**B**) pulmonary embolism (PE, *n* = 164), ^a^ (m: 5.87 (2.22–15.52)*; f: 1.34 (0.52–3.41)); (**C**) superficial vein thrombosis (SVT, *n* = 126). * *p* < 0.001; ** *p* < 0.01; *** *p* < 0.05 (*p* value by Chi-square test). OR, odds ratio; CI, confidence interval.

A					
	GP	DVT	OR (95% CI)	*p* Value for Interaction	
				Age	Sex
**FV R506Q (FVL)**	21 (4.9)	56 (16.3)	3.80 (2.25-4.62) *	0.241	0.169
**FV H1299R (FVR2)**	39 (9.6)	58 (16.9)	1.93 (1.25-2.97) **	0.204	0.856
**FII G20210A**	23 (5.4)	49 (14.3)	2.93 (1.74-4.91) *	0.141	0.548
**B**					
	**GP**	**PE**			
**FV R506Q (FVL)**	21 (4.9)	20 (12.2)	2.71 (1.42-5.14) **	0.649	0.032 ^a^
**FV H1299R (FVR2)**	39 (9.6)	31 (18.9)	2.21 (1.32-3.68) **	0.079	0.171
**FII G20210A**	23 (5.4)	25 (15.2)	3.16 (1.74-5.75) *	0.949	0.698
**C**					
	**GP**	**SVT**			
**FV R506Q (FVL)**	21 (4.9)	22 (17.5)	4.12 (2.18-7.78) *	0.682	0.578
**FV H1299R (FVR2)**	39 (9.6)	25 (19.8)	2.34 (1.35-4.05) **	0.919	0.708
**FII G20210A**	23 (5.4)	14 (11.1)	2.2 (1.09-4.41) ***	0.963	0.372

**Table 4:** Odds ratios and confidence intervals of FV R506Q, FV H1299R and FII G20210A variants. (**A**) Portal venous thrombosis (PVT, *n* = 118), (**B**) cerebral vein thrombosis (CVT, *n* = 75). * *p* < 0.001; ** *p* < 0.01 (*p* value by Chi-square test). OR, odds ratio; CI, confidence interval.

A					
	GP	PVT	OR (95% CI)	*p* Value for Interaction	
				age	sex
**FV R506Q (FVL)**	21 (4.9)	15 (12.7)	2.84 (1.41-5.69) **	0.566	0.567
**FII G20210A**	23 (5.4)	24 (20.3)	4.48 (2.43-8.29) *	0.205	0.131
**B**					
	**GP**	**CVT**			
**FII G20210A**	23 (5.4)	16 (21.3)	4.76 (2.38-9.54) *	0.955	0.179

## Abbreviations

VTE: venous thromboembolism; DVT: deep vein thrombosis; PE: pulmonary embolism: SVT: superficial vein thrombosis; CVT: cerebral vein thrombosis; PVT: portal vein thrombosis; RVT: retinal vein thrombosis; FVL: Factor V Leiden, R506Q variant; FII: Factor II; MTHFR: methylene-tetrahydrofolate reductase; FVR2: Factor V, H1299R variant; FXIII: Factor XIII; HPA: Human Platelet Antigen; PAI: Plasminogen Activator Inhibitor; OR: Odd’s ratio.
